# Medical laboratory practice in Malawi – Current status

**DOI:** 10.4102/ajlm.v12i1.1921

**Published:** 2023-01-06

**Authors:** Symon F. Nayupe, Patrick Mbulaje, Steven Munharo, Parth Patel, Don E. Lucero-Prisno

**Affiliations:** 1 Laboratory Department, Kamuzu University of Health Sciences Private Clinic, Blantyre, Malawi; 2 Center for the Development of People, Lilongwe, Malawi; 3 Montfort Hospital, Chikwawa Diocese, Nchalo, Chikwawa, Malawi; 4 Department of Health Systems and Policy, Faculty of Health Systems, Kamuzu University of Health Sciences, Blantyre, Malawi; 5 Department of Global Health and Development, London School of Hygiene and Tropical Medicine, London, United Kingdom

## Introduction

Medical practice has evolved over the past years from symptom-based clinical diagnoses to evidence-based diagnoses demanding clinical laboratory investigations. Clinical experts at the Mayo Clinic in the United States estimated that almost 70% of patient management decisions rely on laboratory diagnostic information.^1,2^ In sub-Saharan Africa, the need for quality diagnostic services is apparent; nevertheless, access to quality and reliable laboratory services in the region has been a big challenge.^3^

There has been significant development in medical laboratory practice across the world with the adoption of state-of-the-art technology and methods and subsequent demand for more specialised skills in medical laboratory scientists.^4^ In sub-Saharan Africa, medical laboratory practice has also evolved, though significantly slower than in other countries in the West, except countries like the Republic of South Africa which has many accredited and technologically advanced laboratory institutions.^5^

Since many sub-Saharan Africa countries are resource limited, financing of laboratory activities has not been of primary concern; hence, laboratory improvement activities have suffered a persistent shortage of funds, dragging the pace of advancement in medical laboratory practice.^3^ Additionally, the slow growth of medical laboratory practice has also been facilitated by continued neglect of the medical laboratory profession leading to inadequately trained personnel in some parts and an unrecognised cadre.^6^ In Malawi, medical laboratory practice has suffered similar setbacks. The laboratory profession has, nonetheless, achieved some milestones as we will discuss. However, some serious reforms, recommendations of, have to be implemented to tackle the challenges that seriously hamper efficient, high-quality, and technologically advanced diagnostic services as are needed not only in Malawi but in sub-Saharan Africa as a region.

We hereby present the current status of medical laboratory practice in Malawi, from various dimensions, namely human resources, equipment and technology, funding, as well as strengthening policy. As a background, the laboratory is structured in four ascending tiers, namely health centre laboratories, district hospital laboratories, central hospital laboratories and the national reference laboratory.^7^ A lower laboratory refers to the next level laboratory where advanced tests are required. Laboratory falls under the Health Technical Support Services, a directorate under the Ministry of Health. The Health Technical Support Services is responsible for ensuring the provision of quality diagnostic capacity, monitoring drug efficacy, and patient management services.

## Human resources

Malawi is one of the countries in sub-Saharan Africa where the ratio of healthcare workers to patients is high, reflecting a shortage of healthcare staff. Since the 1990s, however, there has been a significant effort from the government to train more healthcare personnel.^8^ Historically, Malamulo College of Health Sciences was the first to offer certificates in medical laboratory sciences in 1968 and later in 1978 started offering diplomas. Since then, Malawi has progressed to offering laboratory science degrees at three accredited institutions namely the University of Malawi, Malawi Adventist University and Mzuzu University today.

As per Malawi Association of Medical Laboratory Scientists (MAMLS) unpublished records for 2021, there were approximately 2069 trained laboratory personnel registered with the Medical Council of Malawi: 677 laboratory technologists, 1073 laboratory technicians and 320 laboratory assistants. Currently, there are over 468 medical laboratory technologists and technicians who are unemployed. A commonly given reason is that there are no posts available as per government establishment despite the country facing a huge gap in laboratory personnel in many facilities. There is no doubt that the laboratory is an essential service, although the practice and utilisation of the service have generally been suboptimal for the past years with an evident need for infrastructural and capacity development.^9^ Good-quality laboratory services are largely dependent on adequate, appropriately trained, and qualified laboratory personnel, yet laboratory professionals are prominently among neglected health cadres in Malawi and across most sub-Saharan African countries.^10^

Services offered are affected by insufficient staff even though colleges are producing many graduates, as there are not enough formal posts currently. In addition, services are affected by lack of specialist qualifications and almost non-existent career progression opportunities since the laboratory profession appears not to be a primary area of concentration for professional improvement and recognition in the country. This understandably lowers the motivation of laboratory professionals. Those employed by the Ministry of Health are often working in underfunded, poorly equipped facilities with low safety standards and unmotivating environments.

## Technology upgrades and investments

The Malawi health sector has gradually expanded and improved tests available to patients. This has happened due to the availability and increased coverage of several modern machines such as the GeneXpert (Cepheid, Sunnyvale, California, United States) and full blood count machines purchased through the Global Fund mainly at district and central hospitals.^11^ In addition, the country has integrated tests on the existing machines to fully utilise the existing technology. For example, targeted viral load testing is now being done on GeneXpert platforms in most facilities, an upgrade from just running tuberculosis specimens. This is a huge investment that has saved money since the procurement is only focusing on procuring the reagents instead of the new machines.

Currently, the laboratory system is still struggling to provide high-quality diagnostic services. Frequent shortages in supplies and reagents challenge sustainable service provision. Additionally, factors such as poor equipment maintenance systems, poor laboratory infrastructure and limited backup testing services exacerbate the operational inefficiencies of testing services. Despite the presence of equipment service contracts for government laboratories in Malawi, the provision of both emergency and routine services for machine breakdowns and maintenance has been significantly slow. This, in part, is due to the availability of a few professionally trained biomedical engineers locally. Consequently, these delays interrupt diagnostic service delivery.^9^

It has been observed that many clinicians often doubt the laboratory results of their patients. This leads to the repetition of tests, since the clinical presentation of the patients is at times inconsistent with results from laboratory investigations.^9^ The observations in this study could be attributed to task shifting, the use of non-laboratory trained personnel to perform tests in point-of-care settings failure to calibrate equipment, usage of expired reagents, lack of external quality control and refresher courses as well as total disregard of laboratory quality management systems.^10^

## Funding and financing

In resource-limited countries, allocation of resources to diagnostic services is barely a priority.^3^ Inadequate funding has downgraded the laboratory profession leading to poor infrastructures failing to meet the demand of its specialty to the growing population. Lack of representation in key decision-making bodies is one of the top contributing factors leading to poor laboratory services in Malawi. This has led to the underperformance of instrument maintenance services and a zero integrated supply chain for laboratory consumables.^11^ The position of laboratory manager is not an established one at the district level and hence it is not represented in the district health management team. Most of the projects in Malawi and sub-Saharan Africa at large are donor driven and hence are somewhat disease specific. This has led to a lack of cross-sector laboratory capacity, fragmentation of laboratory services and diversion of scarce resources.^12^ Poor salary structures have also aggravated the migration of highly skilled individuals to the private sector and research institutions, further derailing the system.

## Strengthening policy

As part of laboratory professional practice improvement, medical laboratory professionals in Malawi have revived its previously dormant body, MAMLS. Formed in 1998, MAMLS was not active until February 2020, when a group of medical laboratory scientists, in collaboration with The International Federation of Clinical Chemistry and Laboratory Medicine, facilitated the hosting of laboratory professionals from across Malawi and guests from Canada, the United States, Egypt and the United Kingdom, to the first ever Medical Laboratory Scientific Conference, where a task force dedicated to revamping MAMLS was formed. In December 2021, a second scientific conference was held in the country’s capital, Lilongwe.^13^

Laboratorians have often complained about the lack of a body specifically formulated to have regulatory oversight over ethical conduct, performing objective quality assurance and accreditation of medical laboratories in the country as well as representing professional interests at the policy level.^14^ The revamping of MAMLS aims at promoting and safeguarding the interests of professional medical laboratory science practice which ultimately safeguards patients who access laboratory services. Continued lobbying by MAMLS focuses on establishing an independent medical laboratory regulatory body that will ensure a robust diagnostic representation at the policy level.

## Recommendations

### Define clear laboratory networks

A strong laboratory organisational infrastructure in sub-Saharan Africa is necessary to improve access to quality healthcare.^10^ Similarly, in Malawi, such a clear definition of function, authority and responsibility of the laboratory system is necessary as a baseline for improving laboratory standards. The Ministry of Health in liaison with the laboratory leadership in Malawi should define these networks. Laboratory networks should include a multilevel systematic integration of functions with an enhanced referral system where laboratories with less testing capacity at the bottom of the system can refer advanced tests to laboratories at higher levels with more testing capacity with ease and within acceptable expected turnaround times. Additionally, laboratories at all levels should adhere to national and international quality systems.^5^ The call for a regulatory body specific for laboratory practice in the strengthening policy section serves this purpose as one of the duties of the body.

### Establish regulatory body for medical laboratories

To curtail challenges faced by laboratories and laboratory personnel to provide quality service, the Malawi government should draft a medical laboratory regulatory act to lead the way in addressing chronic challenges affecting ethical, professional and legal laboratory practice and policy. Establishing a regulatory body will ensure that medical laboratories are objectively regulated for laboratory quality assurance, help shape laboratory policy and improve quality service delivery expected by patients and hospitals. The regulatory act and legal mandate will prevent encroachment and imposition from other departmental mandates.^15^

### Designate specialised laboratory posts

The availability of adequate and trained human resources is one of the key elements to achieving quality diagnostics services. The absence of such, or the presence of staff who have no formally defined roles or positions, compromises the efficiency of achieving such quality as they lack direction and motivation. Designating managerial and non-managerial laboratory-based positions in the medical laboratory setup through the Directorate of Human Resources in Ministry of Health will map out career development prospects as well as equip particular departments with necessary skills depending on individual previous experiences to ensure competence and skills in handling jobs.

### Include medical laboratory professionals in policy boards and regulatory bodies

Including medical laboratory professionals in boards and regulatory bodies by the appointing authorities will ensure the implementation of policy that promotes the welfare of laboratory personnel as well as promotion of the ever-evolving standard quality and harmonised laboratory practice, reshaping regulation and helping to redefine professionalism. Regulation should be done by those vested with the dogma, qualifications, philosophy and understanding of the current problems affecting the profession and trends of medical laboratory science and practice around the globe.

## Conclusion

It is evident that there has been significant progress in the laboratory profession in Malawi and generally in sub-Saharan Africa since colleges started training professionals locally. However, with the current health demands in modern medical practice that require efficient and quality diagnostic services, the laboratory profession is facing new challenges.^15^ Our recommendations on defining clear laboratory networks, enacting a medical laboratory regulatory act, designation of administrative and specialised posts for laboratory professionals at the district and central levels, and the inclusion of laboratory professionals in decision-making bodies will contribute to strengthening laboratory practice in Malawi. Strong laboratory systems will ensure reliable diagnostic services, a contributor to access to quality health services.^10^ Regionally, it is essential to have reliable laboratory systems in sub-Saharan Africa as this not only serves the individual countries but helps to strengthen regional interdependence as countries will now trust each other’s services, leading to the formation of regional networks for advanced and more specialised tests.
